# Spatiotemporal analysis of particulate air pollution and ischemic heart disease mortality in Beijing, China

**DOI:** 10.1186/1476-069X-13-109

**Published:** 2014-12-12

**Authors:** Meimei Xu, Yuming Guo, Yajuan Zhang, Dane Westerdahl, Yunzheng Mo, Fengchao Liang, Xiaochuan Pan

**Affiliations:** Department of Occupational and Environmental Health, School of Public Health, Peking University, Beijing, China; Department of Epidemiology and Biostatistics, School of Population Health, the University of Queensland, Brisbane, Australia; Department of Occupational and Environmental Health, School of Public Health, Ningxia Medical University, Yinchuan, China; Sibley School of Mechanical and Aerospace Engineering, Cornell University, Ithaca, NY USA

**Keywords:** Spatiotemporal analysis, Ischemic heart disease, Particulate matter, Ordinary kriging, Generalized additive mixed model

## Abstract

**Background:**

Few studies have used spatially resolved ambient particulate matter with an aerodynamic diameter of <10 μm (PM_10_) to examine the impact of PM_10_ on ischemic heart disease (IHD) mortality in China. The aim of our study is to evaluate the short-term effects of PM_10_ concentrations on IHD mortality by means of spatiotemporal analysis approach.

**Methods:**

We collected daily data on air pollution, weather conditions and IHD mortality in Beijing, China during 2008 and 2009. Ordinary kriging (OK) was used to interpolate daily PM_10_ concentrations at the centroid of 287 township-level areas based on 27 monitoring sites covering the whole city. A generalized additive mixed model was used to estimate quantitatively the impact of spatially resolved PM_10_ on the IHD mortality. The co-effects of the seasons, gender and age were studied in a stratified analysis. Generalized additive model was used to evaluate the effects of averaged PM_10_ concentration as well.

**Results:**

The averaged spatially resolved PM_10_ concentration at 287 township-level areas was 120.3 ± 78.1 μg/m^3^. Ambient PM_10_ concentration was associated with IHD mortality in spatiotemporal analysis and the strongest effects were identified for the 2-day average. A 10 μg/m^3^ increase in PM_10_ was associated with an increase of 0.33% (95% confidence intervals: 0.13%, 0.52%) in daily IHD mortality. The effect estimates using spatially resolved PM_10_ were larger than that using averaged PM_10_. The seasonal stratification analysis showed that PM_10_ had the statistically stronger effects on IHD mortality in summer than that in the other seasons. Males and older people demonstrated the larger response to PM_10_ exposure.

**Conclusions:**

Our results suggest that short-term exposure to particulate air pollution is associated with increased IHD mortality. Spatial variation should be considered for assessing the impacts of particulate air pollution on mortality.

**Electronic supplementary material:**

The online version of this article (doi:10.1186/1476-069X-13-109) contains supplementary material, which is available to authorized users.

## Background

Ischemic heart disease (IHD) is one of the most common causes of death worldwide, causing 7,249,000 deaths in 2008, 12.7% of total global mortality [1]. According to Global Burden of Disease Study in 2010, the number of ischemic heart disease deaths rose from 450.3 million in 1990 to 948.7 million in 2010, ranking the second leading causes of death in China in 2010 [2]. A number of risk factors for ischemic heart disease have been suggested, such as age, gender, hypertension, obesity and smoking [1–3].

Some studies have indicated that exposure to air pollution was associated with IHD mortality [4–6], morbidity [7], and hospital admissions [8, 9]. Studies on the impacts of ambient particles less than 10 μm in aerodynamic diameter (PM_10_) on health have also been performed in China, but most have used non-spatial data of daily PM_10_, e.g. monitoring values from one station or the average concentrations of a limited number of monitor stations, to estimate the association between PM_10_ and IHD mortality [10–12]. This may result in inaccuracies, specifically exposure misclassification, as PM_10_ may vary over a specific area due to expected differences in PM_10_ levels by the impact of local sources and meteorology. It is not clear how this uncertainty would impact risk estimations, either toward overestimation or underestimation, but it does make such evaluations much more difficult [13–15].

Various techniques (e.g. inverse distance weighting, land use regression analysis and geo-statistical methods such as kriging) have been developed to interpolate air pollution values at the locations where data are unavailable using data collected at multiple sites [16, 17]. Some studies have applied the interpolation methods to examine the health effects of air pollution in studies [18, 19]. There also has been evidence that the methods used to generate estimates of exposure to air pollution could affect the health risk estimates in epidemiological studies [20, 21].

Studies have applied interpolation methods to estimate the air pollutant concentrations in China [22, 23]. However, there is no study that has used spatially resolved PM_10_ concentrations to quantify the impact of PM_10_ on IHD mortality in China. The goal of this research is to apply a generalized additive mixed model to examine the association between spatially resolved PM_10_ concentrations and IHD mortality in Beijing.

## Methods

### Study area

Beijing is the capital of China, and located in the northern tip of the roughly triangular North China Plain. It has an area of 16,410 square kilometers, with 14 urban and suburban administrative districts (Dongcheng, Xicheng, Chaoyang, Haidian, Changping, Fengtai, Shijingshan, Mentougou, Daxing, Fangshan, Tongzhou, Shunyi, Huairou and Pinggu District) and two rural counties (Minyun and Yanqing County) which included 304 township-level areas. The population is 1.96 million (The Sixth National Population Census, Beijing, 2010). For townships, the size ranges from 1 to 390 square kilometers and the population ranges from 2000 to 359400 (The Sixth National Population Census, Beijing, 2010). It has a dry, monsoon-influenced humid continental climate, characterized by hot, humid summers and cold, windy, dry winters. Average annual temperature and precipitation was 14.0°C and 483.9 mm, respectively. Ambient air pollution is seriously elevated along with the increasing of fuel consumption (including vehicles, power plants and industries) and construction projects in the city.

### Data collection

Daily numbers of IHD deaths between 1 January 2008 and 31 December 2009 were obtained from China Centers for Disease Control and Prevention (China CDC) for 287 township-level areas. The IHD death data were unavailable in Minyun and Yanqing Counties. The deaths at each area were residents of the corresponding area. IHD was defined according to the International Classification of Diseases, 10^th^ version (ICD-10:I20-I25). The data were classified by gender (female and male) and age (<65 and ≥65 years).

**Figure 1:**
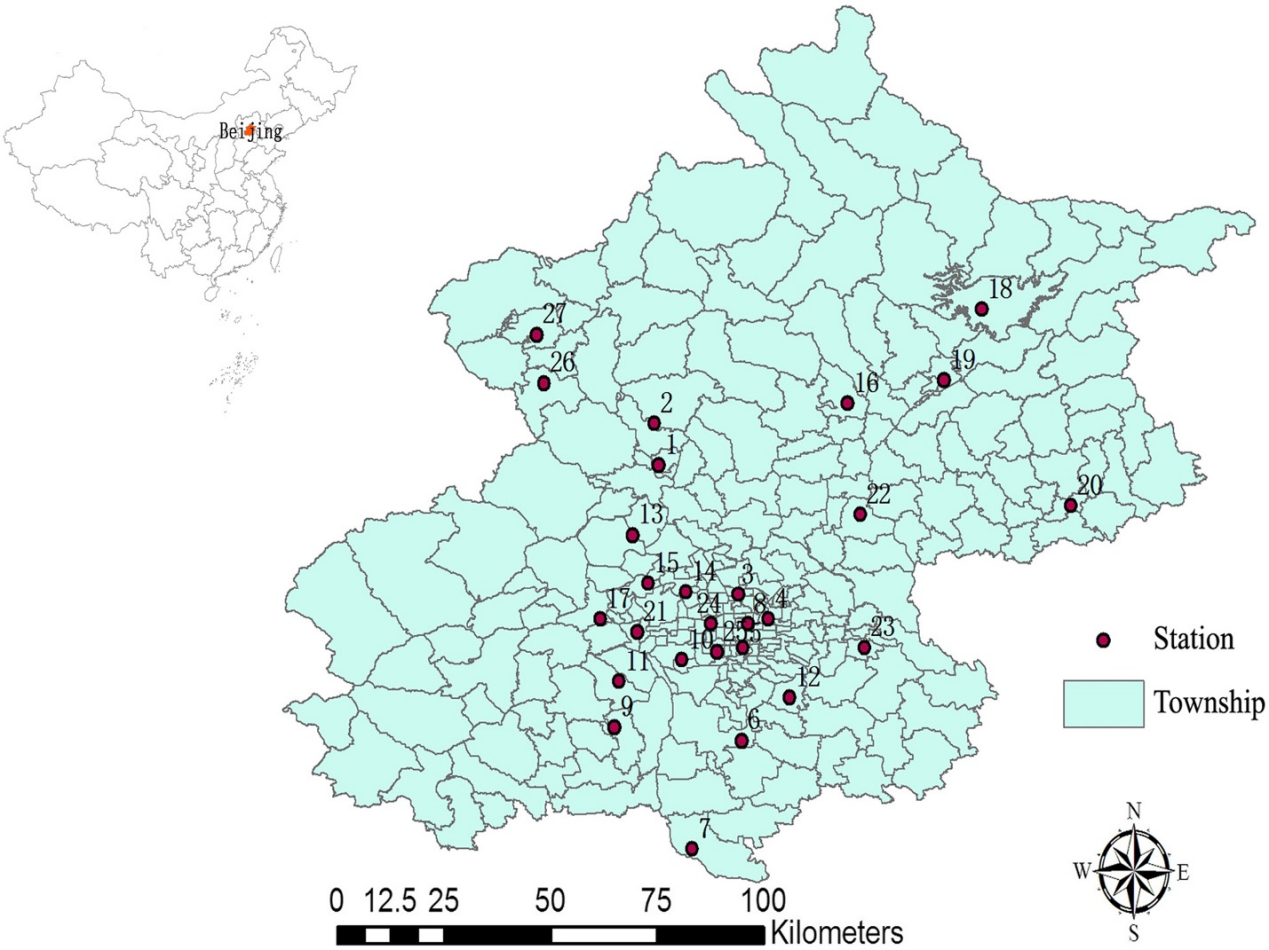
**The 27 monitoring stations for PM**_**10**_ **in Beijing.**

PM_10_ data of 27 ambient air quality monitoring sites in Beijing city were collected from the Beijing Municipal Environmental Protection Bureau (Figure 1). The missing rate during the study period was from 0.4% to 6.7%. Imputation will produce error so we did not fill the missing value before interpolating. For SO_2_/NO_2_, only a single daily average concentration for the whole city was available from the Beijing Public Net for Environmental Protection. To control for the effect of weather conditions on IHD mortality, daily meteorological data on mean temperature and relative humidity from one station (located at N39°48′ E116°28′) were obtained from China Meteorological Data Sharing Service System.

### Data analysis

#### Spatial interpolation for PM_10_ concentration

We selected two methods, inverse distance weighting (IDW) and ordinary kriging (OK), to interpolate the daily PM_10_ concentrations from the values of 27 monitoring sites to the centroids of the 304 township-level areas across Beijing city. IDW and kriging are the most common interpolation methods. About the performance of IDW and kriging, the findings have been mixed [24].

The IDW interpolation method is to estimate the value of a given location by a weighted average of data at nearby monitors, where interpolation weights for each monitor’s value are computed as a function of distance between observed sample sites and the site to be predicted [16]. We used λ_i_ = 1/d_i_^2^ as a weighting factor for the monitor site i, where d_i_ is the distance between the monitor site i and the point to be predicted (i.e., the centroid of each township).

The kriging method is a geo-statistical technique and also a weighted combination of monitor values that uses spatial autocorrelation among data to determine the weights [16]. OK is the most common kriging method. It assumes a constant but unknown mean, which allows construction of an unbiased estimator that does not require prior knowledge of the stationary mean of the observed values [23]. In this study, we estimated the data at the centroid of each township using OK.

To test the validity of the interpolation methods and provide a more quantitative comparison of the two models, we conducted "leave-one-out cross-validation" (LOOCV). This method involves using a single monitor values as the validation data and the remaining monitor values as the training data. This is then repeated such that each monitor is used once as the validation data. The difference (including the root-mean-square error (RMSE) and the mean) and the correlation between the observed and predicted values were calculated as the measure indices of LOOCV.

#### Modeling the association between PM_10_ and IHD mortality

A generalized addictive mixed model (GAMM) was applied to analyze the effects of PM_10_ on IHD mortality, which uses additive nonparametric functions to formulate covariate effects and adds random effects to the additive predictor accounting for over-dispersion and correlation [25, 26]. We put the township-level IHD deaths as the dependent variable and the corresponding township-level PM_10_ estimates as the main independent variable in GAMM. Penalized Quasi-likelihood method [27, 28], accounting for the over-dispersion of daily death counts, was used in GAMM framework to model the natural logarithm of the expected daily death counts as a function of the predictor variables. A random area-level intercept in GAMM can be used to model those areas with higher death rates [29, 30].

First, the basic model was built excluding the air pollution variables. The penalized spline functions of time and weather variables for accommodating nonlinear relationships of mortality with these variables were incorporated. The partial autocorrelation function was used to guide the selection of degrees of freedom (df) for time trend [31]. We used squared Pearson scaled residuals to compare the fit of the models [30]. In this way, a penalized spline with seven degrees of freedom per year for time trend, which had the smallest sum of the absolute partial autocorrelation values over a 30-day lag period, was used to control for the seasonal and long-term trends. Because temperature’s effects on health may be lagged for more than 10 days [32, 33], the 14-day moving average temperature was controlled in our model [34, 35]. The present-day relative humidity was incorporated in the models because no evidence of confounding by this variable was shown in air pollution epidemiology [35]. Three degrees of freedom for temperature and relative humidity were chosen based on the model fitting [36]. The day of the week (DOW) and public holiday (PH) was adjusted as a categorical variable in the basic model. After the basic model was established, the pollutant variables were introduced. The final model was:


Where i is the township (township = 1, ……, 287); t is the day; Y_i,t_ is the number of IHD deaths in township i on day t. α is the intercept. PM10_i,t_ is the daily PM_10_ concentration in township i on day t. S(.) is a penalized spline; Temp_t_ is the mean temperature on day t; RH_t_ is relative humidity on day t; a spline with 3 degrees of freedom (df) was used for temperature and relative humidity. A spline with 7 degrees of freedom per year for time was used to control for season and long-term trend. DOW_t_ is the categorical variable day of the week on day t. PH_t_ is the indicator of public holiday on day t. Z_i_ is a random intercept for township i.

In order to compare the effects of spatial resolved PM_10_ and averaged PM_10_ of 27 monitoring stations in Beijing, we also used GAMM and generalized additive model (GAM) to examine the impacts of averaged PM_10._ The same confounders as GAMM were adjusted in GAM as follows:


We next analyzed the associations between PM_10_ and IHD mortality with different lag structures, i.e. single-day lags (from lag 0 to lag 5) and multiday lags (lag 0–1 to 0–5). In single-day lag models, lag 0 referred to the current-day air pollutants concentration, and lag 1 corresponded to the previous-day concentration; while in multiday lag models, lag 0–1 meant the 2-day moving average concentration of current day and the previous day.

Single and multiple air pollutant models were also fitted to examine the effects of PM_10_ on IHD mortality in GAMM. In the single-pollutant model, PM_10_ was put alone in the model; in the two-pollutant models, SO_2_ (lag0-1) or NO_2_ (lag0-1)) were jointly included. SO_2_ or NO_2_ at lag 0–1 was controlled because this lag was shown to be more strongly associated with health effects [37, 38].

Stratified analyses by gender, age and season also were conducted. For season—spring, summer, autumn and winter are defined as March–May, June–August, September–November and December–February, respectively. The Z test was used to detect statistically differences between effect estimates from stratified analyses [39].

Sensitivity analyses were conducted to check the impacts of PM_10_ on IHD mortality using the different degrees of freedom (4–9) for time trend, temperature (4–6) and relative humidity (4–6) as well as controlling 14-day moving average relative humidity in the model. All the sensitivity analyses were done only for the whole population.

All the data analysis was performed in statistical software R version 3.0.1 (R Development Core Team, 2013). The "gstat" package was used to interpolate spatial PM_10_. The "mgcv" package was used to fit GAMM and GAM. All statistical tests were two-sided and P-values with less than 0.05 were considered statistically significant. The results are presented as the percent change and 95% confidence intervals (95%CIs) in daily IHD mortality per 10-μg/m^3^ increase in PM_10_ concentrations.

## Results

Table 1 showed the descriptive statistics for IHD deaths, air pollutants and weather data in Beijing during the study period. There were a total of 26,653 IHD deaths (14,240 males and 12,413 females) from Jan 1 2008 to Dec 31 2009. The average daily deaths of IHD were about 40, with the most occurring in the winter months and the least in the summer, of which 17.7% were for <65 years old and 82.3% for ≥ 65 years old.

**Table 1:** **Summary statistics for PM**_**10**_ **, spatially resolved PM**_**10**_ **, SO**_**2**_ **, NO**_**2**_ **, daily mean temperature, daily mean relative humidity and daily IHD death counts in Beijing between 2008 and 2009**

	Season	Min	25%	Median	75%	Max	Mean	SD
PM_10_(μg/m^3^)*^a^	Spring	7.0	80.0	124.0	184.0	600.0	144.5	96.3
	Summer	5.0	64.0	98.0	134.0	463.2	101.4	52.2
	Autumn	7.0	54.0	94.0	148.0	553.0	110.1	75.9
	Winter	7.0	62.0	108.0	170.0	600.0	127.7	88.7
	Overall	5.0	64.0	104.0	150.0	600.0	120.8	81.6
Spatially resolved PM_10_(μg/m^3^)^b^	Spring	13.1	84.9	125.4	186.7	593.1	147.6	93.4
	Summer	17.3	69.0	98.8	1338	432.0	103.7	50.4
	Autumn	11.3	61.3	99.1	150.8	489.9	114.9	75.1
	Winter	13.2	61.6	96.9	144.4	600.0	114.9	78.8
	Overall	11.3	68.0	104.2	150.7	600.0	120.3	78.1
SO_2_(μg/m^3^)	Spring	6	14.0	25.0	39.8	138	30.7	22.7
	Summer	6	8.0	11.0	14.0	44	12.5	6.7
	Autumn	6	12.0	16.0	29.0	136	24.9	21.8
	Winter	10	39.3	72.0	102.0	202	75.7	42.3
	Overall	6	12.0	21.0	46.0	202	36.0	35.6
NO_2_(μg/m^3^)	Spring	16	41.6	49.6	59.2	152	51.6	18.4
	Summer	14.4	28.8	40.0	44.8	62.4	37.9	11.0
	Autumn	19.2	40.0	52.8	67.2	142.4	58.2	26.6
	Winter	9.6	40.0	59.2	80.6	140.8	60.6	25.9
	Overall	9.6	36.8	48.0	60.8	152	52.2	23.2
T(°C)	Spring	-0.1	10.0	15.6	20.6	26.8	15.2	6.5
	Summer	17.5	24.3	26.0	28.0	31.6	26.0	2.7
	Autumn	-2	7.1	15.1	19.9	25.4	13.4	7.6
	Winter	-9.4	-3.5	-1.5	0.5	9.4	-1.3	3.4
	Overall	-9.4	2.3	15.4	23.55	31.6	13.4	11.1
RH(%)	Spring	15	30.0	44.0	56.0	95	44.8	18.2
	Summer	19	57.0	68.0	76.0	90	64.8	15.5
	Autumn	19	44.0	60.5	71.0	88	57.2	18.0
	Winter	11	24.0	38.0	53.0	82	39.8	17.4
	Overall	11	35	53	68	95	51.7	19.9
IHD(N)	Spring	20	32.8	37	42	57	37.3	7.3
	Summer	19	28	32	37	49	32.5	6.4
	Autumn	18	34	40	46	67	41.0	9.8
	Winter	29	43	49	55	75	49.1	8.4
	Overall	18	33	39	46	75	39.9	10.1

Note: PM_10_: Particulate matter with an aerodynamic diameter of <10 μm; SO_2_: sulfur dioxide; NO_2_: nitrogen dioxide; T: Temperature; RH: Relative humidity; IHD: Ischemic heart disease; N: Number of death.

*The maximum limit of detection for PM_10_ concentration is 600 μg/m^3^.

^a^Representing the average of the 27 monitoring stations.

^b^Representing the average of the 287 townships where health data was available.

The average temperature and relative humidity were 13.4 ± 11.1°C and 51.7 ± 19.9% during the study period. The mean values of SO_2_ and NO_2_ were 36.0 μg/m^3^ and 52.2 μg/m^3^, respectively. The air pollution levels varied across seasons. The mean daily SO_2_ level was higher in winter and spring than in autumn and summer while the mean daily NO_2_ level was higher in winter and autumn than in spring and summer.

In summary, the mean daily PM_10_ concentrations were 120.8 ± 81.6 μg/m^3^. There were higher PM_10_ concentrations in spring and winter than in autumn and summer. At 27 monitoring stations, the daily means of PM_10_ ranged from 72.6 μg/m^3^ to 144.9 μg/m^3^; 67.9% ~25.7% days had higher PM_10_ level than the China ambient air quality standard level-II (150 μg/m^3^) (Additional file 1: Table S1).The correlations in daily PM_10_ concentrations between stations were strong (Additional file 1: Table S2).

To estimate PM_10_ concentration more precisely, we adopted "leave-one-out" cross-validations to provide a more quantitative comparison of the interpolation methods (Additional file 1: Table S3). Every index of cross-validations indicated OK gave more accurate spatial PM_10_ estimates than IDW. The estimated PM_10_ using OK were strongly correlated with observed PM_10_, with the correlation coefficient ranging from 0.90 to 0.99 (P < 0.01). Generally, the differences between observed and spatially resolved PM_10_ were small.

**Figure 2:**
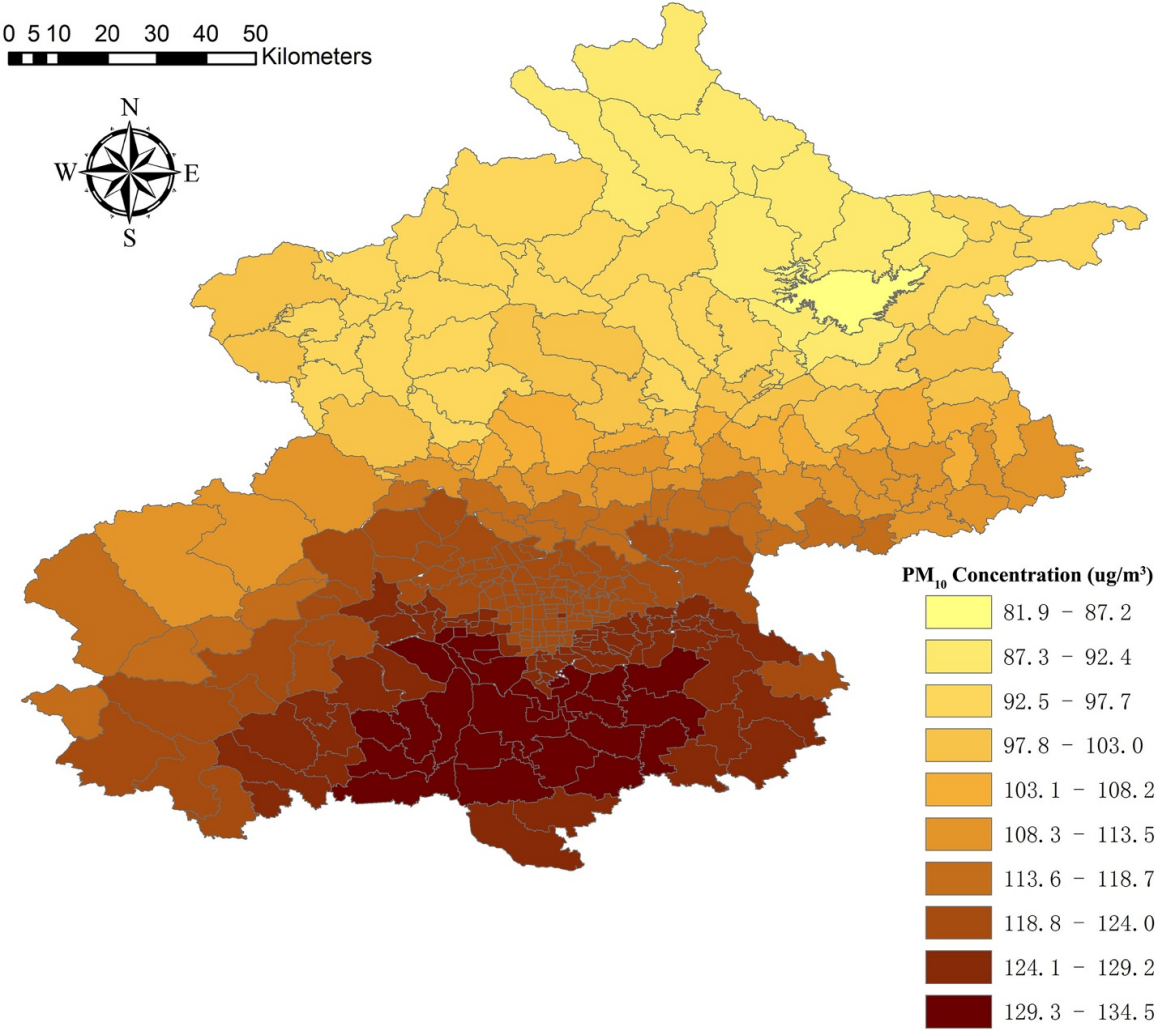
**The averaged spatially resolved PM**_**10**_ **concentrations at 304 towns in Beijing during 2008–2009.**

The averaged spatially resolved PM_10_ concentration at 287 township-level areas was 120.3 ± 78.1 μg/m^3^, following the same trend in seasons as the observed PM_10_ levels. During the study period, the average daily PM_10_ concentration in the south of Beijing was higher than that in the north of Beijing (Figure 2).

The Spearman correlations between air pollutants and meteorological variables during the whole study were presented (Additional file 1: Table S4). PM_10_ was positively associated with other air pollutants and meteorological variables. The correlation between PM_10_ and NO_2_ (r = 0.55) was stronger than that between PM_10_ and SO_2_ (r = 0.43).

**Figure 3:**
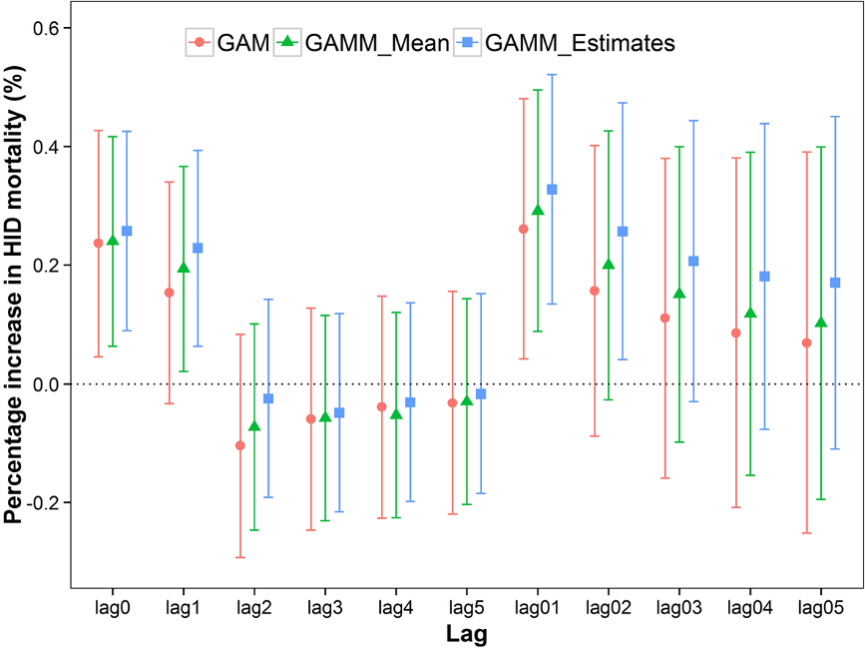
**Percentage increase of IHD mortality associated with a 10-μg/m** ^**3**^ **increase in PM**_**10**_ **concentration in Beijing, China.** Note: GAM: Estimated effects in GAM using averaged PM_10_; GAMM_Mean: Estimated effects in GAMM using averaged PM_10_; GAMM_Estimates: Estimated effects in GAMM using spatially resolved PM_10_.

Figure 3 shows the association between PM_10_ and IHD mortality using spatially resolved PM_10_ concentrations. We observed statistically significant associations of daily IHD mortality with PM_10_ on the current day (lag 0), the previous day (lag 1), the moving average 2 days (lag 0–1) and the moving average 3 days (lag 0–2). We estimated an increase of 0.26% (95% CI: 0.09%, 0.43%), 0.23% (95% CI: 0.06%, 0.39%), 0.33% (95% CI: 0.13%, 0.52%) and 0.26% (95% CI: 0.04%, 0.47%) in IHD mortality associated with a 10-μg/m^3^ increase in PM_10_ at lag 0, lag 1, lag 0–1 and lag 0–2, respectively. The largest effects was observed for 2-day average.

For the effects of averaged PM_10_ on IHD mortality, we also observed the largest effects at lag 0–1 using GAMM and GAM. However, the effect estimates were smaller and the confidence intervals were larger than those using spatially resolved PM_10_ (Figure 3).

**Figure 4:**
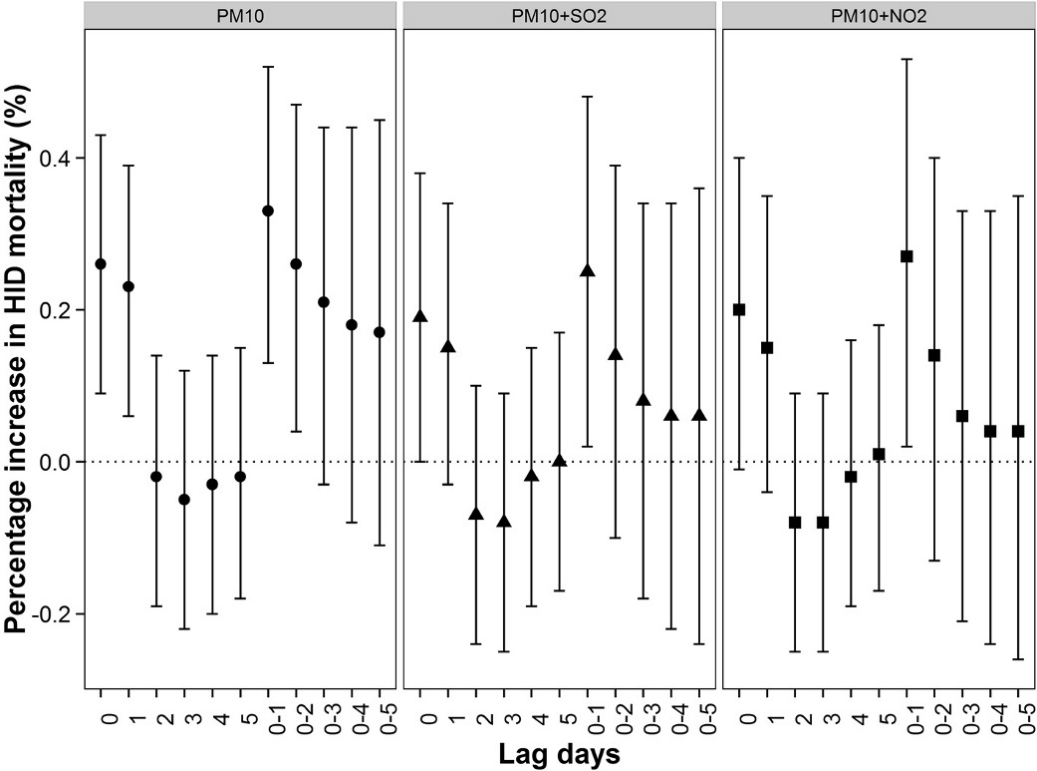
**Percent increase in IHD mortality associated with a 10-μg/m** ^**3**^ **increase in PM**_**10**_ **concentrations using the single- and two-pollutant models in GAMM.**

In the two-pollutant model, the association of PM_10_ with IHD mortality was seen to be reduced at all lag patterns after adjustment for SO_2_ or NO_2_ (Figure 4), but still remained significant at lag 0–1 day.

The associations between PM_10_ and IHD mortality differed by season (Table 2). The effects of PM_10_ on IHD mortality were the strongest in summer, with a 10-μg/m^3^ increase associated with a 0.83% (95% CI: 0.31%, 1.35%), 0.88% (95% CI: 0.31%, 1.45%) increase of IHD mortality at lag 0–1 and lag 0–2 days, respectively. The differences of effect estimates between summer and spring as well as between summer and winter were statistically significant.

**Table 2:** **Percentage increase in IHD mortality associated with a 10-μg/m** ^**3**^ **increase in PM**_**10**_ **concentrations by four seasons using the single-pollutant model in GAMM**

Lag	Season	Percent change (95% CI)
lag0	Spring	0.17(-0.07,0.40)
	Summer	0.60(0.13,1.06)*
	Autumn	0.35(0.06,0.64)*
	Winter	0.23(-0.06,0.51)
lag1	Spring	0.19(-0.05,0.43)
	Summer	0.68(0.22,1.14)*^b^
	Autumn	0.27(-0.01,0.56)
	Winter	0.12(-0.16,0.41)
lag2	Spring	0.03(-0.21,0.27)
	Summer	0.43(-0.03,0.90)^b^
	Autumn	-0.08(-0.37,0.21)
	Winter	-0.16(-0.44,0.12)
lag01	Spring	0.24(-0.02,0.51)
	Summer	0.83(0.31,1.35)*^a^
	Autumn	0.41(0.08,0.73)*
	Winter	0.27(-0.06,0.60)
lag02	Spring	0.22(-0.06,0.51)
	Summer	0.88(0.31,1.45)*^ab^
	Autumn	0.29(-0.06,0.65)
	Winter	0.14(-0.22,0.51)

Note: *P < 0.05.

^a^The difference of effect estimate between summer and spring was statistically significant (p < 0.05).

^b^The difference of effect estimate between summer and winter was statistically significant (p < 0.05).

CI: confidence interval.

**Figure 5:**
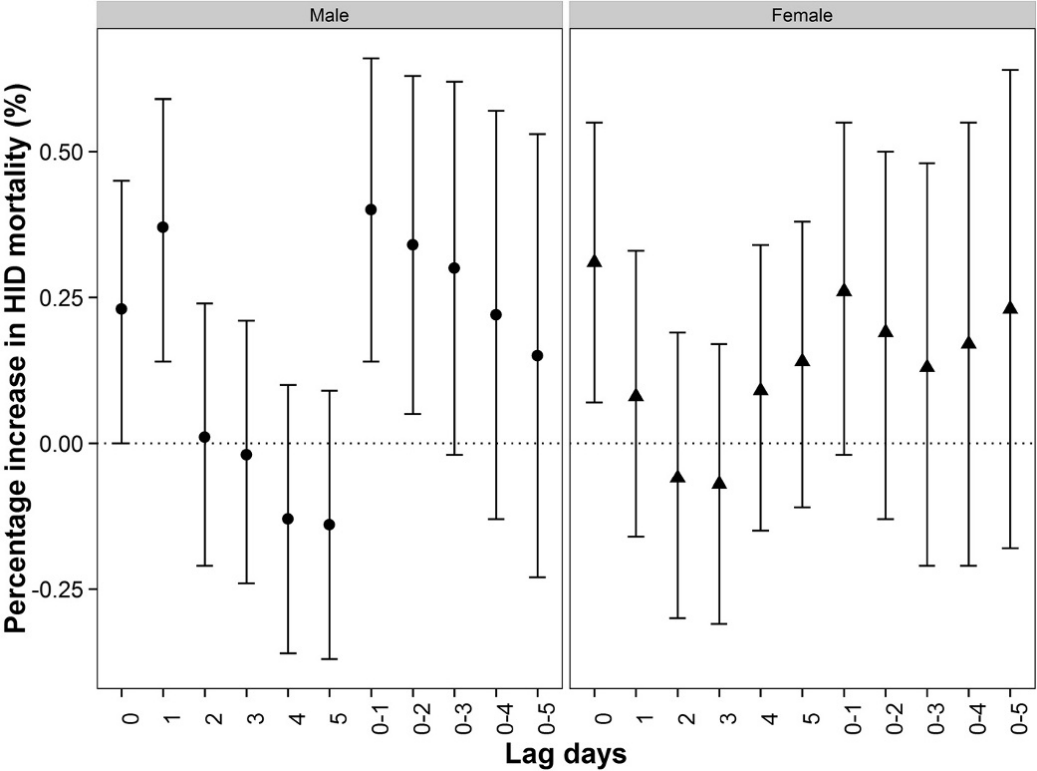
**Percent increase (95% CI) in IHD mortality associated with a 10-μg/m** ^**3**^ **increase in PM**_**10**_ **concentrations by sex using the single-pollutant model in GAMM.**

**Figure 6:**
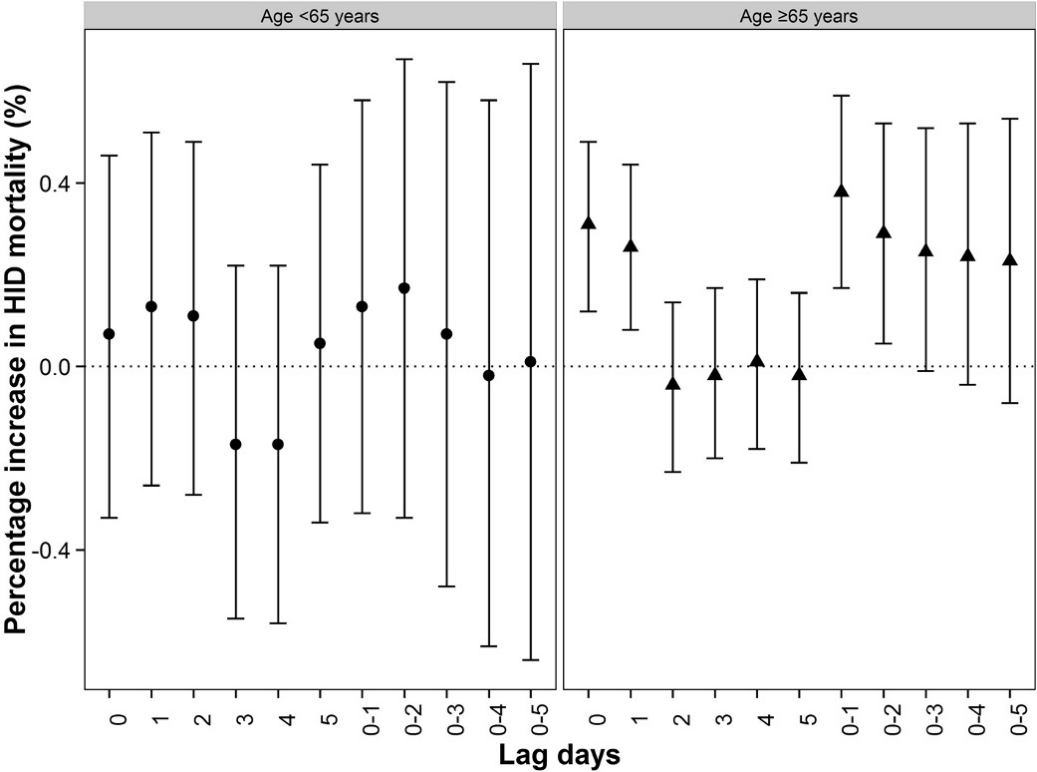
**Percent increase (95% CI) in IHD mortality associated with a 10-μg/m** ^**3**^ **increase in PM**_**10**_ **concentrations by age using the single-pollutant model in GAMM.**

The association of PM_10_ with IHD mortality also varied by gender and age group (Figures 5 and 6). The largest effects of PM_10_ were observed on the current day for females and at lag 0–1 for males. The effect estimates of PM_10_ among females were higher than those among males on the current day while the effect estimates of PM_10_ among males were higher than those among females at lag 1 day and lag 0–1 days. However, the between-gender differences were not statistically significant. We observed the effect estimates among people aged ≥65 years were significant and approximately 3 times higher than those aged < 65 years at lag 0–1, but the differences of effect estimates between age groups were not statistically significant.

Sensitivity analysis was conducted to check our findings. Changing the degrees of freedom for time, temperature and relative humidity did not substantially affect the association of PM_10_ with IHD mortality. The effects estimates were hardly changed when 14-day moving average relative humidity was controlled in our model. These results suggested that our findings are statistically robust.

## Discussion

We found that there were statistically significant associations between spatially resolved estimated PM_10_ mass concentration and increased risk of IHD death of the exposed population in Beijing, China. To our knowledge, it is the first time to use the spatiotemporal analysis method to examine the acute effects of ambient PM_10_ on IHD mortality, and also the first study to show spatial variation of ambient PM_10_ level in township-level of Beijing. We also examined whether the effect estimates varied by age, gender and season.

Studies [40, 41] have shown that the concentrations of air pollutants varied spatially across a specific area. Capturing the spatial variation using spatial modeling methods have been used to estimate air pollutants values from multiple monitor stations to the whole study region or exposure at the individual level. However, there was no consistent conclusions on which method was the best. Air pollution exposure estimates using spatial methods are affected by several factors, including the density and location of monitors and the available variables affecting air pollutants concentrations. Firstly, governmental monitor stations usually are placed in urban region, while fewer monitors are available in rural areas. This may result in misestimating the exposure in rural areas when using the values from nearby urban areas. Secondly, industrial and traffic emissions that may be more important in the urban areas, land use patterns and meteorological factors can influence the spatial and temporal distribution of air pollutants. Some models, such as land use regression model (LUR) [24, 42] and generalized addictive mix model (GAMM) [43–45] allowing for those variables, have been utilized to estimate the air pollutants exposure. Studies showed LUR or GAMM performed better than the conventional spatial interpolation methods (inverse distance weighting, nearest neighbor method or kriging) [43–47]. Those variables were unavailable in our study, so we are left only to estimate PM_10_ using the simple spatial interpolation methods. We found that the OK produced more accurate and less biased estimates than inverse distance weighting based on cross-validation results; therefore, we applied OK to interpolate PM_10_ concentrations over each township in Beijing.

Particulate matter may trigger ischemic heart disease through several possible mechanisms, including increasing inflammation [48], abnormal regulation of cardiac autonomic system [49], increasing blood viscosity [50] and vasoconstrictor such as endothelins [51]. Previous studies have reported inconsistent association between PM_10_ and ischemic heart disease mortality [10, 11, 52]. In our analysis, the largest effect was observed for 2-day average, with a 0.33% (95% CI: 0.13%, 0.52%) increase of IHD mortality per 10-μg/m^3^ increase of 2-day moving average PM_10_. The magnitude of our estimates was smaller than previous findings [10, 11] . For example, Li et al. [11] found IHD mortality increased by 0.53% (95% CI: 0.30%, 0.84%) for a 10 μg/m^3^ increment PM_10_ on the same day in Tianjin. However, in Netherlands, Hoek et al. [52] did not observed statistically significant association between PM_10_ and IHD mortality. The heterogeneity of these findings may be explained by the different characteristics of the study sites such as PM_10_ level, components of PM_10_, sensitivity of local residents to PM_10_, indoor air pollution, weather patterns [53, 54]. In addition, the df selection decisions and the different lag patterns in GAM and the number of study years could affect the estimated effects [55, 56].

Our study observed harvesting effects although the effects have no statistically significance. This means that PM_10_ may hasten the deaths of persons who were extremely frail. But the effect sizes of PM_10_ rebound because PM_10_ could exacerbate ischemic heart disease [57], potentially increasing the number of sensitive persons whose illness is life threatening.

Consistent with previous reports [15, 58], we found that the effect estimates using spatially resolved PM_10_ were larger than that using averaged PM_10_ from multiple stations. This suggested that previous time-series studies using the average levels may underestimate the effects of PM_10_. Although the effects difference using different exposure metrics was not too large, it still needs be considered especially in cities with large spatial variation of air pollutants and cannot be ignored because the association between air pollution and mortality itself was weak.

In the two-pollutant models, the associations between PM_10_ and IHD mortality adjusting for SO_2_ or NO_2_ were attenuated and become insignificant at some lag patterns, which may be caused by the collinearity between PM_10_ and NO_2_ as well as SO_2_ (Additional file 1: Table S4). The findings are consistent with previous studies [58]. So far it is still an unresolved scientific question to separate the independent effects of individual air pollutant from multiple-pollutant models in short-term effects studies of air pollution. Moreover, in order to better examine the effects of spatial resolved PM_10_ in multiple-pollutant model, the other air pollutants also needed to be estimated spatially because between-pollutant relationships may not be characterized well just by the averaged value in one area. Further studies are needed to resolve these problems.

Seasonal differences in the short-term effects of PM_10_ on IHD mortality were found in this study. The association in the summer period was stronger than in the other seasons. Li et al. [11] also identified the strongest effects of PM_10_ on IHD mortality in summer in Tianjin, China. However, Chen et al. [59] observed the largest estimates of PM_10_ on daily mortality in winter and summer in northern cities of China which have similar meterological conditions to Beijing. There are several explanations for the inconsistent findings in the studies. Firstly, the particulate matter constituents may vary by season in these cities. We cannot obtain the data of the PM_10_ components, which hinders further study on how the different particulate matter constituents affect the effects by season. In additional, socioeconomic characteristics, activity patterns of local residents and statistical models used could partially account for the discrepant finding.

We found the effect estimate of PM_10_ on IHD mortality in females was larger than those in males on the current day. This suggested that females were more sensitive to PM_10_, which was possibly due to higher airway hyper-responsiveness to oxidants, more deposition of fine particles or relatively lower socioeconomic status [54]. The larger lag effects in males may be partly explained by the higher incidence of heart disease in males than in females, particularly pre-menopausal females. Studies have shown that biological factors, such as hormone levels, help protect women against heart disease [60].

Our study also found the elderly were more susceptible to PM_10_ exposure than the younger group. This is consistent with previous reports [54, 61]. Preexisting chronic disease such as cardiorespiratory disease in the elderly are more prevalent than in the younger group.

This study has several limitations. Firstly, we cannot obtain daily data on SO_2_, NO_2_, temperature and relative humidity from multiple stations, so the township-based spatial distributions of the covariates cannot be estimated. Consequently, we did not control for the spatial variation of these variable in our model, which may result in a bias in effect estimates. Secondly, our exposure assignment approach assumed that the subjects lived and worked at the same township, and considered outdoor air concentrations at the centroid of the corresponding township as personal exposure, which might result in exposure misclassification. Thirdly, studies have shown that exposure measurement error may affect the effect estimations when exposure predictions as explanatory variables are incorporated into a regression model for health effects analyses [62, 63]. The exposure measurement error contains a Berkson-like component that increases the variance of the effect estimate and a classical-type component that not only increase the variance but also bias the effect estimates [64]. Szpiro and his colleagues developed a method for measurement error correction based on asymptotic approximations that derived for linear regression for the exposure and health models [64, 65]. To date, there has been no methods for measurement error correction used in the nonlinear regression models and assessing health effects of multiple predicted pollutants exposure [65]. Thus, we did not correct the measurement error in our study, which may have an impact on the effect estimates.

## Conclusions

Ambient PM_10_ concentration was statistically significant associated with IHD mortality of the population in spatiotemporal analysis in Beijing, China. The stronger association occurred for the 2-day average. Season, gender and age appear to modify the effects of PM_10_ on IHD mortality. GAMM considering spatial variations of ambient PM_10_ produced greater effect estimates than GAM using averaged PM_10_ concentrations. It implies that spatial variation should be considered for assessing the impacts of air pollution on mortality. Our findings may have implication for primary prevention of IHD deaths in China and guiding future work on more advanced methods of estimated exposure and health effects.

## Electronic supplementary material

Additional file 1: Table S1: Summary statistics for daily PM_10_ (μg/m^3^) at 27 monitoring stations in Beijing, China between 2008 and 2009 (see Figure 1 for the locations). Table S2 Spearman correlations between daily PM_10_ concentrations at 27 monitoring stations in Beijing city between 2008 and 2009. Table S3 The comparison between the predicted and observed PM_10_ concentrations using different interpolation methods at 27 monitoring stations during 2008-2009. Table S4 The correlation between pollutants and meteorological variables. (DOC 164 KB)

## Abbreviations

PM_10_: Particulate matter with an aerodynamic diameter of <10 μm
GAM: Generalized additive model
GAMM: Generalized additive mixed model
CI: Confidence interval
ICD-10: International classification of diseases 10th version
CDC: China centers for disease control and prevention
SO_2_: Sulfur dioxide
NO_2_: Nitrogen dioxide
T: Temperature
RH: Relative humidity
SD: Standard deviation
*df*: Degree of freedom
IDW: Inverse distance weighting
OK: Ordinary Kriging
LOOCV: Leave-one-out cross-validation
DOW: Day of the week
RMSE: Root-mean-square error.
