# Supplementary material for: Spatiotemporal analysis of particulate air pollution and ischemic heart disease mortality in Beijing, China
Source: Environ Health. 2014 Dec 12;13:109. doi: 10.1186/1476-069X-13-109 (PMC4293109; doi:10.1186/1476-069X-13-109)
Supplement: Supplementary file 1 — Additional file 1: Table S1: Summary statistics for daily PM10 (μg/m3) at 27 monitoring stations in Beijing, China between 2008 and 2009 (see Figure 1 for the locations). Table S2 Spearman correlations between daily PM10 concentrations at 27 monitoring stations in Beijing city between 2008 and 2009. Table S3 The comparison between the predicted and observed PM10 concentrations using different interpolation methods at 27 monitoring stations during 2008-2009. Table S4 The correlation between pollutants and meteorological variables. (DOC 164 KB) [file 12940_2014_817_MOESM1_ESM.doc]

**Supplementary Material**

Table S1 Summary statistics for daily PM_10_ (μg/m^3^) at 27 monitoring stations in Beijing, China between 2008 and 2009 (see Figure 1 for the locations)

Table S2 Spearman correlations between daily PM_10_ concentrations at 27 monitoring stations in Beijing city between 2008 and 2009

Table S3 The comparison between the predicted and observed PM_10_ concentrations using different interpolation methods at 27 monitoring stations during 2008-2009

Table S4 The correlation between pollutants and meteorological variables

**Table S1**

**Summary statistics for daily PM_10_ (****μg/m^3^) at 27 monitoring stations in Beijing, China between 2008 and 2009 (see Figure 1 for the locations)**

| Station | Min | 25% | Median | 75% | Max* | Mean | SD | P(%) |
| --- | --- | --- | --- | --- | --- | --- | --- | --- |
| 1 | 11 | 58 | 95 | 138 | 600 | 109.4 | 74.0 | 45.7 |
| 2 | 7 | 45 | 78 | 126 | 600 | 93.9 | 72.5 | 35.1 |
| 3 | 12 | 70 | 110 | 162 | 600 | 128.3 | 84.1 | 55.5 |
| 4 | 15 | 68 | 108 | 150 | 600 | 125.0 | 83.1 | 54.2 |
| 5 | 15 | 68 | 104 | 156 | 600 | 123.1 | 78.8 | 52.2 |
| 6 | 17 | 86 | 123 | 180 | 600 | 144.7 | 88.7 | 65.5 |
| 7 | 13 | 74 | 108 | 160 | 600 | 127.0 | 82.0 | 54.2 |
| 8 | 11 | 74 | 116 | 162 | 600 | 132.0 | 84.8 | 59.1 |
| 9 | 17 | 88 | 126 | 176 | 600 | 144.9 | 87.1 | 67.9 |
| 10 | 15 | 83.5 | 122 | 178 | 600 | 139.6 | 84.9 | 63.7 |
| 11 | 12 | 73.5 | 114 | 166 | 600 | 129.9 | 81.2 | 57.9 |
| 12 | 14 | 80 | 120 | 180 | 600 | 140.4 | 88.7 | 61.2 |
| 13 | 18 | 78 | 120 | 174 | 600 | 133.8 | 83.0 | 59.6 |
| 14 | 12 | 74 | 112 | 160 | 600 | 128.9 | 82.2 | 58.4 |
| 15 | 7 | 58 | 98 | 150 | 600 | 114.6 | 78.3 | 48.8 |
| 16 | 9 | 50.5 | 86 | 130 | 600 | 99.5 | 68.5 | 41.5 |
| 17 | 12 | 74 | 118 | 166.5 | 600 | 131.3 | 81.5 | 60.3 |
| 18 | 5 | 25 | 56 | 102 | 600 | 72.6 | 62.6 | 25.7 |
| 19 | 7 | 48 | 84 | 130 | 600 | 98.4 | 71.0 | 39.7 |
| 20 | 7 | 60 | 98 | 146 | 600 | 114.6 | 79.7 | 47.7 |
| 21 | 10 | 78 | 124 | 174 | 600 | 136.9 | 82.7 | 63.1 |
| 22 | 11 | 68 | 108 | 158 | 600 | 123.2 | 82.3 | 53.6 |
| 23 | 10 | 80 | 116 | 166 | 600 | 134.8 | 81.8 | 60.7 |
| 24 | 12 | 64 | 102 | 148.5 | 600 | 119.0 | 79.7 | 51.0 |
| 25 | 12 | 68 | 108 | 159 | 600 | 123.4 | 80.2 | 54.7 |
| 26 | 11 | 42 | 74 | 126 | 600 | 92.8 | 73.0 | 34.7 |
| 27 | 12 | 52 | 84 | 134 | 600 | 98.8 | 66.6 | 40.3 |

Note: Min: Minimum; Max: Maximum; SD: Standard deviation; P: Percentage of daily PM_10_ value above Chinese ambient air quality standard level-II (150 μg/m^3^)

*:The maximum limit of detection for PM10 concentration is 600 μg/m3

**Table S2**

Spearman correlations between daily PM_10_ concentrations at 27 monitoring stations in Beijing city between 2008 and 2009 *^a^*

|  | 1 | 2 | 3 | 4 | 5 | 6 | 7 | 8 | 9 | 10 | 11 | 12 | 13 | 14 | 15 | 16 | 17 | 18 | 19 | 20 | 21 | 22 | 23 | 24 | 25 | 26 |
| --- | --- | --- | --- | --- | --- | --- | --- | --- | --- | --- | --- | --- | --- | --- | --- | --- | --- | --- | --- | --- | --- | --- | --- | --- | --- | --- |
| 2 | 0.95 |  |  |  |  |  |  |  |  |  |  |  |  |  |  |  |  |  |  |  |  |  |  |  |  |  |
| 3 | 0.93 | 0.89 |  |  |  |  |  |  |  |  |  |  |  |  |  |  |  |  |  |  |  |  |  |  |  |  |
| 4 | 0.91 | 0.87 | 0.98 |  |  |  |  |  |  |  |  |  |  |  |  |  |  |  |  |  |  |  |  |  |  |  |
| 5 | 0.91 | 0.86 | 0.97 | 0.97 |  |  |  |  |  |  |  |  |  |  |  |  |  |  |  |  |  |  |  |  |  |  |
| 6 | 0.81 | 0.76 | 0.89 | 0.90 | 0.93 |  |  |  |  |  |  |  |  |  |  |  |  |  |  |  |  |  |  |  |  |  |
| 7 | 0.83 | 0.77 | 0.88 | 0.89 | 0.91 | 0.92 |  |  |  |  |  |  |  |  |  |  |  |  |  |  |  |  |  |  |  |  |
| 8 | 0.91 | 0.87 | 0.97 | 0.98 | 0.98 | 0.92 | 0.89 |  |  |  |  |  |  |  |  |  |  |  |  |  |  |  |  |  |  |  |
| 9 | 0.85 | 0.81 | 0.92 | 0.92 | 0.94 | 0.93 | 0.90 | 0.93 |  |  |  |  |  |  |  |  |  |  |  |  |  |  |  |  |  |  |
| 10 | 0.88 | 0.83 | 0.94 | 0.95 | 0.95 | 0.94 | 0.89 | 0.96 | 0.94 |  |  |  |  |  |  |  |  |  |  |  |  |  |  |  |  |  |
| 11 | 0.91 | 0.87 | 0.95 | 0.95 | 0.95 | 0.90 | 0.89 | 0.96 | 0.94 | 0.95 |  |  |  |  |  |  |  |  |  |  |  |  |  |  |  |  |
| 12 | 0.83 | 0.77 | 0.91 | 0.92 | 0.94 | 0.96 | 0.92 | 0.94 | 0.93 | 0.95 | 0.92 |  |  |  |  |  |  |  |  |  |  |  |  |  |  |  |
| 13 | 0.94 | 0.90 | 0.93 | 0.92 | 0.92 | 0.87 | 0.86 | 0.92 | 0.90 | 0.92 | 0.94 | 0.88 |  |  |  |  |  |  |  |  |  |  |  |  |  |  |
| 14 | 0.92 | 0.88 | 0.96 | 0.96 | 0.96 | 0.91 | 0.90 | 0.96 | 0.93 | 0.96 | 0.96 | 0.93 | 0.95 |  |  |  |  |  |  |  |  |  |  |  |  |  |
| 15 | 0.94 | 0.94 | 0.95 | 0.93 | 0.92 | 0.82 | 0.81 | 0.93 | 0.88 | 0.90 | 0.94 | 0.84 | 0.93 | 0.95 |  |  |  |  |  |  |  |  |  |  |  |  |
| 16 | 0.95 | 0.93 | 0.90 | 0.89 | 0.90 | 0.80 | 0.83 | 0.89 | 0.84 | 0.86 | 0.89 | 0.82 | 0.92 | 0.90 | 0.92 |  |  |  |  |  |  |  |  |  |  |  |
| 17 | 0.91 | 0.87 | 0.94 | 0.94 | 0.95 | 0.90 | 0.88 | 0.95 | 0.94 | 0.94 | 0.96 | 0.92 | 0.94 | 0.96 | 0.93 | 0.88 |  |  |  |  |  |  |  |  |  |  |
| 18 | 0.83 | 0.88 | 0.83 | 0.80 | 0.79 | 0.67 | 0.63 | 0.81 | 0.74 | 0.75 | 0.80 | 0.70 | 0.79 | 0.79 | 0.87 | 0.90 | 0.80 |  |  |  |  |  |  |  |  |  |
| 19 | 0.92 | 0.91 | 0.91 | 0.90 | 0.91 | 0.83 | 0.83 | 0.91 | 0.87 | 0.88 | 0.90 | 0.85 | 0.90 | 0.90 | 0.91 | 0.96 | 0.90 | 0.93 |  |  |  |  |  |  |  |  |
| 20 | 0.91 | 0.90 | 0.91 | 0.91 | 0.91 | 0.84 | 0.86 | 0.90 | 0.87 | 0.88 | 0.90 | 0.85 | 0.90 | 0.90 | 0.91 | 0.94 | 0.90 | 0.87 | 0.94 |  |  |  |  |  |  |  |
| 21 | 0.91 | 0.86 | 0.95 | 0.94 | 0.95 | 0.90 | 0.87 | 0.95 | 0.93 | 0.94 | 0.95 | 0.91 | 0.94 | 0.96 | 0.93 | 0.88 | 0.96 | 0.80 | 0.90 | 0.89 |  |  |  |  |  |  |
| 22 | 0.92 | 0.90 | 0.95 | 0.94 | 0.94 | 0.84 | 0.83 | 0.94 | 0.88 | 0.90 | 0.92 | 0.87 | 0.90 | 0.93 | 0.93 | 0.94 | 0.92 | 0.88 | 0.95 | 0.94 | 0.92 |  |  |  |  |  |
| 23 | 0.82 | 0.77 | 0.91 | 0.92 | 0.94 | 0.94 | 0.90 | 0.93 | 0.92 | 0.93 | 0.90 | 0.95 | 0.87 | 0.91 | 0.83 | 0.82 | 0.91 | 0.73 | 0.87 | 0.87 | 0.91 | 0.90 |  |  |  |  |
| 24 | 0.93 | 0.89 | 0.98 | 0.98 | 0.98 | 0.92 | 0.89 | 0.98 | 0.94 | 0.97 | 0.97 | 0.94 | 0.94 | 0.98 | 0.95 | 0.91 | 0.96 | 0.82 | 0.92 | 0.92 | 0.96 | 0.95 | 0.92 |  |  |  |
| 25 | 0.91 | 0.87 | 0.96 | 0.97 | 0.99 | 0.93 | 0.91 | 0.98 | 0.94 | 0.96 | 0.96 | 0.94 | 0.93 | 0.97 | 0.93 | 0.90 | 0.95 | 0.80 | 0.91 | 0.92 | 0.96 | 0.94 | 0.93 | 0.98 |  |  |
| 26 | 0.91 | 0.93 | 0.84 | 0.82 | 0.82 | 0.73 | 0.76 | 0.81 | 0.77 | 0.78 | 0.82 | 0.74 | 0.85 | 0.83 | 0.89 | 0.88 | 0.82 | 0.80 | 0.84 | 0.83 | 0.81 | 0.84 | 0.73 | 0.83 | 0.82 |  |
| 27 | 0.88 | 0.88 | 0.84 | 0.83 | 0.83 | 0.79 | 0.77 | 0.84 | 0.82 | 0.83 | 0.85 | 0.80 | 0.91 | 0.87 | 0.86 | 0.87 | 0.86 | 0.79 | 0.87 | 0.85 | 0.85 | 0.84 | 0.80 | 0.86 | 0.85 | 0.88 |

*^a^* All the correlations were statistically significant (P<0.01)

**Table S3**

The comparison between the predicted and observed PM_10_ concentrations using different interpolation methods at 27 monitoring stations during 2008 and 2009

| station | Ordinary kriging | | | |  | Inverse distance weighted | | | |
| --- | --- | --- | --- | --- | --- | --- | --- | --- | --- |
|  | Correlation^a^ | RMSE | Mean | SD |  | Correlation^a^ | RMSE | Mean | SD |
| 1 | 0.98 | 14.45 | -0.83 | 14.44 |  | 0.98 | 15.50 | 0.08 | 15.51 |
| 2 | 0.97 | 20.96 | -12.07 | 17.15 |  | 0.98 | 18.68 | -9.99 | 15.80 |
| 3 | 0.99 | 14.74 | 5.18 | 13.8 |  | 0.99 | 14.88 | 4.90 | 14.05 |
| 4 | 0.99 | 12.96 | -3.63 | 12.45 |  | 0.99 | 13.82 | -3.98 | 13.24 |
| 5 | 0.99 | 12.87 | -5.19 | 11.79 |  | 0.99 | 12.83 | -5.16 | 11.75 |
| 6 | 0.97 | 24.14 | 9.59 | 22.16 |  | 0.97 | 21.41 | 5.66 | 20.66 |
| 7 | 0.93 | 30.38 | -7.74 | 29.4 |  | 0.94 | 43.96 | -30.67 | 31.52 |
| 8 | 0.99 | 13.62 | 7.64 | 11.28 |  | 0.99 | 13.97 | 7.60 | 11.72 |
| 9 | 0.96 | 27.38 | 14.12 | 23.47 |  | 0.96 | 24.25 | 7.62 | 23.03 |
| 10 | 0.97 | 22.26 | 11.97 | 18.78 |  | 0.98 | 20.62 | 10.63 | 17.69 |
| 11 | 0.97 | 20.22 | -6.56 | 19.14 |  | 0.97 | 21.65 | -8.21 | 20.05 |
| 12 | 0.97 | 22.66 | 7.81 | 21.29 |  | 0.97 | 21.55 | 5.89 | 20.74 |
| 13 | 0.96 | 29.31 | 18.16 | 23.03 |  | 0.96 | 29.79 | 18.39 | 23.45 |
| 14 | 0.98 | 15.73 | 4.86 | 14.97 |  | 0.98 | 16.10 | 5.24 | 15.23 |
| 15 | 0.96 | 24.67 | -13.1 | 20.91 |  | 0.96 | 26.17 | -14.07 | 22.08 |
| 16 | 0.97 | 20.83 | -10.58 | 17.95 |  | 0.97 | 17.16 | -4.90 | 16.46 |
| 17 | 0.98 | 16.91 | 3.93 | 16.46 |  | 0.98 | 16.60 | 1.98 | 16.50 |
| 18 | 0.90 | 42 | -29.27 | 30.14 |  | 0.90 | 34.74 | -17.90 | 29.79 |
| 19 | 0.98 | 15.25 | -0.89 | 15.23 |  | 0.98 | 14.83 | 3.99 | 14.29 |
| 20 | 0.94 | 26.8 | 4.33 | 26.47 |  | 0.93 | 30.69 | 8.37 | 29.54 |
| 21 | 0.97 | 20.13 | 7.7 | 18.61 |  | 0.97 | 20.48 | 7.83 | 18.94 |
| 22 | 0.98 | 20.82 | 7.92 | 19.27 |  | 0.98 | 21.25 | 9.55 | 19.00 |
| 23 | 0.95 | 25.11 | 6.48 | 24.28 |  | 0.96 | 24.04 | 4.31 | 23.66 |
| 24 | 0.99 | 12.14 | -8.39 | 8.79 |  | 0.99 | 12.59 | -8.59 | 9.21 |
| 25 | 0.99 | 12.96 | -4.96 | 11.98 |  | 0.99 | 13.18 | -4.96 | 12.22 |
| 26 | 0.93 | 28.26 | -11.93 | 25.63 |  | 0.94 | 26.36 | -9.31 | 24.68 |
| 27* | 0.92 | 26.95 | 1.63 | 26.92 |  | 0.90 | 33.40 | 10.46 | 31.75 |
| overall | 0.96 | 22.4 | -0.14 | 22.4 |  | 0.96 | 24.49 | -1.58 | 24.44 |

Note: ^a^ All the Pearson correlations were statistically significant (p<0.01)

RMSE: Root-mean-square error; SD: Standard deviation

**Table S4**

The correlation between pollutants and meteorological variables^a^

|  | spatially resolved PM_10_ | SO_2_ | NO_2_ | T | RH |
| --- | --- | --- | --- | --- | --- |
| spatially resolved PM_10_ | 1 |  |  |  |  |
| SO_2_ | 0.43 | 1 |  |  |  |
| NO_2_ | 0.55 | 0.67 | 1 |  |  |
| T | 0.06 | -0.67 | -0.33 | 1 |  |
| RH | 0.21 | -0.24 | 0.11 | 0.4 | 1 |

Note: *^a^* All the correlations were statistically significant (P<0.01)

T: Temperature; RH: Relative Humidity
